# Long-Term Storage Does Not Affect the Expression Profiles of mRNA and Long Non-Coding RNA in Vitrified-Warmed Human Embryos

**DOI:** 10.3389/fgene.2021.751467

**Published:** 2022-02-01

**Authors:** Jingyu Li, Ling Zhu, Jin Huang, Weiwei Liu, Wei Han, Guoning Huang

**Affiliations:** ^1^ Chongqing Key Laboratory of Human Embryo Engineering, Chongqing Reproductive and Genetics Institute, Chongqing Health Center for Women and Children, Chongqing, China; ^2^ Information Department, Chongqing Health Center for Women and Children, Chongqing, China

**Keywords:** human embryo, vitrification, storage time, single-cell RNA-seq, lncRNA

## Abstract

Although vitrification has been widely applied in assisted reproductive technology, it is unknown whether storage time has any impact on the mRNA and lncRNA expression profiles in human embryos. Eleven women (aged 23–35 years) who had undergone *in vitro* fertilization treatment were recruited for this study. The transcriptomes of 3 fresh eight-cell embryos and 8 surviving vitrified-warmed eight-cell embryos (4 embryos were cryostored for 3 years, and the others were cryostored for 8 years) were analyzed through single-cell RNA-Seq. No differentially expressed mRNAs or lncRNAs were identified between the 3-years group and 8-years group. A total of 128 mRNAs and 365 lncRNAs were differentially expressed in the 8 vitrified-warmed embryos compared with the fresh embryos. The vitrification-warming impact was moderate, and it was mainly related to the pathways of metabolism, stress response, apoptosis, cell cycle, cell adhesion, and signaling for TFG-β and Hippo. The analysis of target mRNAs suggested that lncRNAs might contribute to the regulation of mRNAs after vitrification-warming. Our findings indicated that long-term storage after vitrification does not affect the mRNA and lncRNA expression profiles in human embryos, however, the procedure of vitrification-warming would lead to minor alteration of transcriptome.

## Introduction

Since the first successful pregnancy from frozen embryos was reported in 1983, embryo cryopreservation has been widely used for over 30 years (Trounson and Mohr, 1983). The application of cryopreservation technology allows multiple embryo transfers from a single stimulation cycle, which improves the cumulative live birth rate (Pandian et al., 2005; Zhu et al., 2018). In addition, frozen embryo transfer reduces the risk of ovarian hyperstimulation syndrome, and minimizes the multiple pregnancy rates by decreasing the number of transferred embryos (Jones et al., 1995; Wirleitner et al., 2013).

Slow freezing and vitrification have been adopted as two principal cryopreservation methods (Edgar and Gook, 2012). Compared with slow freezing, vitrification is a fast cryopreservation method that allows solidification of the cell(s) and extracellular milieu into a glass-like state, thus preventing the formation of ice crystals and cell damage (Rienzi et al., 2017). Recently, several studies have demonstrated that vitrification can significantly increase the survival rate, clinical pregnancy rate, and live birth rate compared with slow freezing (AbdelHafez et al., 2010; Edgar and Gook, 2012). Owing to the improvement of clinical outcomes with vitrification, many laboratories from worldwide have completely replaced slow freezing with vitrification procedure.

During the process of vitrification, embryos are first exposed to a high concentration of cryoprotectants, and then frozen by direct contact with liquid nitrogen. Therefore, many concerns about the potential effect of cryoprotectants and liquid nitrogen contact have been raised (Gosden, 2011; Kopeika et al., 2015). Recently, clinical data indicated that children born from frozen embryo transfer had a significantly higher birthweight than those conceived by fresh embryo transfer (Li et al., 2020a; Huo et al., 2020). Moreover, evidence from human and animal studies demonstrated that vitrification has effects on the expression of embryonic genes involved in development, metabolism, stress response, and apoptosis (Wang et al., 2010; Shaw et al., 2012; Zhao et al., 2013; Gupta et al., 2017). For example, vitrified mouse embryos exhibited differential apoptotic genes, including Bax, Bcl2, and p53 (Glujovsky et al., 2016; Glujovsky and Farquhar, 2016). Specifically, a study in vitrified–warmed human embryos identified expression alteration of seven genes related to apoptosis and pluripotency (Shaw et al., 2012). Generally, most *in vitro* fertilization (IVF) centers perform vitrification of human embryos on day 3 (six-to eight-cell stage). This period coincides with zygotic genome activation (ZGA), which corresponds to the greatest alteration in gene expression, and subsequently affects development (Xue et al., 2013; Yan et al., 2013).

With the large application of embryo cryopreservation in assisted reproductive technology (ART), both the number and the storage time of cryopreserved embryos have been increased. Therefore, the safety of long-term cryopreservation of embryos needs further evaluation. Few studies explored the influence of long-term storage time on human embryos, and these studies have only considered embryo survival, and pregnancy outcomes (Riggs et al., 2010; Liu et al., 2014; Li et al., 2020b; Hamazaki et al., 2020). However, no data regarding the effect of storage time on embryonic gene expression profiles are available right now.

In this study, we aimed to explore the effect of the length of storage time on the mRNA and lncRNA expression profiles of vitrification cryopreserved human eight-cell embryos. For this purpose, we compared the transcriptomes of vitrified-warmed human embryos after 3 and 8 years of cryopreservation by using single-cell RNA-Seq. The transcriptomes of fresh human eight-cell embryos were also examined and used as a control.

## Materials and Methods

### Ethics Statement

This study was approved by the Institutional Review Board (IRB) of Chongqing Health.

Center for Women and Children (2018-RGI-02). We followed the guiding principles from the Ministry of Science and Technology (MOST) for the review and approval of human genetic resources. All donor couples voluntarily donated embryos after signing written informed consent at the Chongqing Reproductive and Genetics Institute in the Chongqing Health Center for Women and Children.

### Patient Selection, Treatment and Oocyte Retrieval

A total of 11 women who received IVF treatment, ≤ 35 years old (range: 23–35 years), without a history of genetic diseases or smoking were included in this study.

Pituitary downregulation and controlled ovarian stimulation were carried out as previously described (Glujovsky and Farquhar, 2016). Briefly, after downregulation with a gonadotrophin-releasing hormone (GnRH) agonist (Triptorelin Acetate, Ipsen Pharma, France), the ovaries were stimulated with recombinant FSH (rFSH) (Puregon; Organon, Netherlands or Gonal-F, Merck Serono, Switzerland). Human chorionic gonadotropin (HCG) (Ovidrel, Merck Serono, Italy) was administered when at least three follicles measured >18 mm. Transvaginal oocyte retrieval was performed 36 h after HCG injection. Cumulus-enclosed oocytes were collected in 2.5 ml IVF medium (G-IVF, Vitrolife Sweden AB, Gothenburg, Sweden) and incubated in 5% O_2_, 6% CO_2_, and 37°C incubators for insemination.

### Embryo Culture

We placed the fertilized oocytes into a pre-equilibrated culture dish (Thermo Scientific, Waltham, MA, United States) with 25 µL of culture droplets (Vitrolife Sweden AB, Gothenburg, Sweden) covered with 1.2 ml of paraffin oil (Vitrolife Sweden AB, Gothenburg, Sweden). The embryos were cultured in an incubator (MCO-5M; Sanyo, Osaka, Japan) at 37°C with 5% O_2_ and 6% CO_2_ until embryo vitrification on day 3.

### Embryo Vitrification and Warming

Vitrification was performed using a commercial kit (Kitazato Company, Japan), in accordance with a previous report (Xiong et al., 2016). First, embryos were transferred to equilibration solution for 12–15 min. Then, the embryos were exposed to the vitrification solution for 45–60 s. Finally, embryos were loaded on the tip of a Cryotop with a small volume of vitrification solution and immersed in liquid nitrogen immediately.

Embryos warming was performed with a four-step protocol. First, vitrified embryos on the tip of Cryotop were dipped into 1.0 M sucrose solution (TS), which had been preheated to 37°C for 2 h, and kept there for 1 min. Second, the embryos were suspended in 0.5 M sucrose solution (DS) for 3 min and then in WS1 for 5 min and WS2 for 1 min. Finally, the embryos were transferred to medium for culture.

### Embryo Collection

The embryos were cultured for 4 h after warming, briefly exposed to an acidic PBS solution for 5–10 s to remove the zona pellucida, and washed three times in PBS. After all the embryos in one group were prepared, we removed them from PBS, and immediately placed each embryo per tube into lysis buffer.

### RNA-Seq Library Generation

We performed amplification using the Smart-Seq2 method. We used the Qubit® 3.0 Fluorometer and Agilent 2100 Bioanalyzer to check the quality of the cDNA product and to ensure that its length was approximately 1–2 kb. The library was prepared following the manufacturer’s instructions (Illumina. Cat. FC-131–1024). After library preparation, we checked the library quality using the PerkinElmer LabChip® GX Touch and Step OnePlus™ Real-Time PCR System. The libraries were then sequenced on the Illumina HiSeq 4000 with a 150-bp paired-end.

### RNA-Seq Data Processing

We used Trim_Galore to remove raw sequence reads that contained adapter contamination and poor-quality reads with low PHRED scores. HISAT (version 2.2.0) was used to map the clean reads to the human reference genome (GRCh38).

For mRNAs, FeatureCounts (version 2.0.1) was used to calculate the read count of genes based on the GRCh38.100 annotation file which was downloaded from the Ensembl database. Differential expression analysis was performed using the DESeq2 (version 1.30.0) package in the R project, and mRNAs with significantly higher changes (absolute value of log2 (fold changes) ≥ 2 and false discovery rates (FDR) < 0.05) were considered differentially expressed mRNAs (DEmRNAs).

For lncRNAs, the annotation file was downloaded from LNCipedia (Version 5.2). FeatureCounts (version 2.0.1) was used to calculate the read count of lncRNAs based on this annotation file. Differential expression analysis was performed using the DESeq2 (version 1.30.0) package in the R project, and lncRNAs with significantly higher changes (absolute value of log2 (fold changes) ≥ 2 and false discovery rates (FDR) < 0.05) were considered differentially expressed lncRNAs (DElncRNAs).

### Enrichment Analysis

We performed gene ontology (GO) analysis and Kyoto Encyclopedia of Genes and Genomes (KEGG) pathway analysis using Metascape webtools. For the GO analysis, we chose Biological Processes terms as the background gene sets. For the KEGG analysis, KEGG Pathway were chosen as the background gene sets. The annotations with a *p* value <0.05 and counts >3 were considered significant.

Gene Set Enrichment Analysis (GSEA) was performed by the GSEA software (version 4.1.0, http://www.gsea-msigdb.org/gsea/downloads.jsp). Gene Ontology (GO) Biological Processes were chosen as the background gene sets, and the threshold of significance was defined as a *p* value <0.05.

### Prediction of *Cis*- and Trans-Target Genes of lncRNAs

To classify lncRNA *cis*-target mRNAs, the BEDTools (version 2.29.2) software was used to search for protein-coding genes located within 10 kb upstream and downstream of the DElncRNAs. To identify the *trans*-target genes of DElncRNAs, a two-step method was conducted. In Step 1, the cor.test () function in the R project was used to calculate the Pearson correlation coefficient between the expression of DElncRNAs and mRNAs. LncRNA-mRNA pairs with |cor| > 0.7 and *p* value <0.05 were retained. In Step 2, the LncTar (version 1.0) software was used to predict the potential interactions between the lncRNA and mRNA sequences, with the parameters “-d -0.2 -s T.” Finally, a merged result was constructed by intersecting the result from Step 1 and the result from Step 2.

## Results

### Deep Sequencing of Different Human Embryos

To investigate the potential effects of storage time after vitrification on the transcriptomes of human embryos, we performed single-cell RNA-Seq on eleven donated human eight-cell embryos from three groups: fresh embryos, cryopreserved embryos stored for 3 years, and cryopreserved embryos stored for 8 years. RNA libraries showed acceptable quality in all embryos. The mean number of raw reads was 43.63 ± 3.23 (±SD) million, and the average mapping rate is 94% (Supplementary Table S1).

### Effect of Storage Time on the mRNA Expression Profiles in Vitrified-Warmed Human Embryos

**FIGURE 1:**
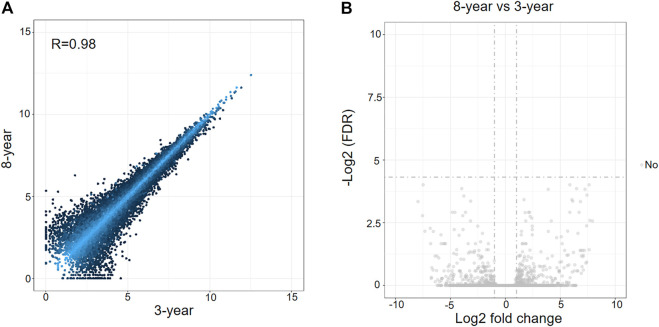
Analysis of differentially expressed mRNAs between 3-years group and 8-years group. **(A)** Scatterplot of mRNA expression variation between the two groups. The normalized expression values of each gene are shown on the x-axis and the y-axis. **(B)** Volcano plots of mRNA expression variation between the two groups. Gray dots represent non-differentially expressed genes between the two groups.

The expression levels of mRNAs in the two groups of surviving vitrified-warmed embryos after 3 and 8 years of storage were compared. However, the mRNA expression profiles of the two groups showed low variation (Figure 1A). Furthermore, no differentially expressed mRNAs (DEmRNAs) were identified between the two groups (Figure 1B).

### Distinct mRNA Expression Profiles From Fresh and Frozen Human Embryos

**FIGURE 2:**
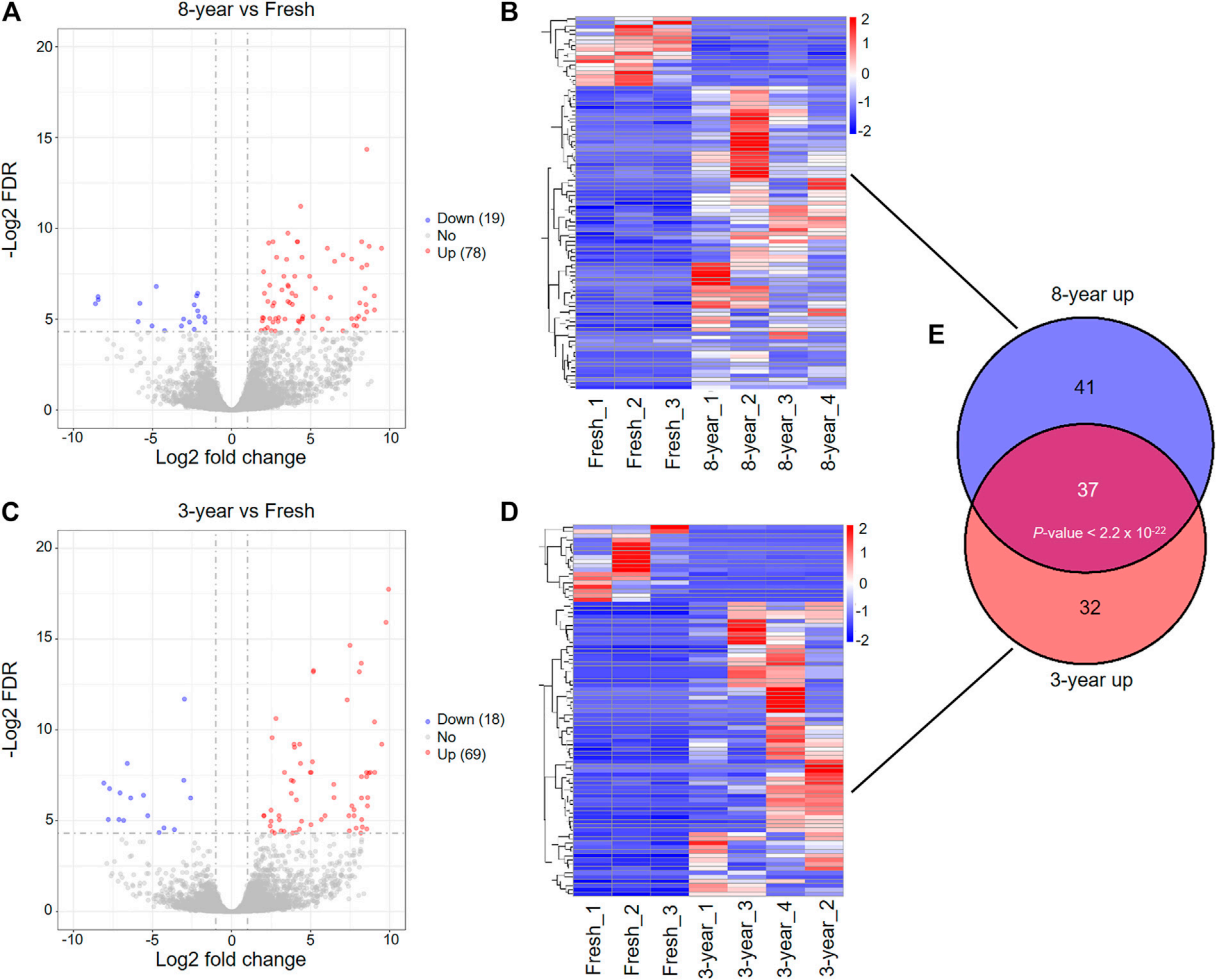
Differentially expressed mRNAs between fresh embryos and vitrified-warmed embryos with different storage times. **(A)** Volcano plots of the DEmRNAs between the 8-years group and fresh group. Red and blue dots represent upregulated and downregulated genes in the 8-years group. Gray dots represent non-differentially expressed genes. **(B)** Heatmap showing the expression profile of DEmRNAs between the 8-years group and fresh group. **(C)** Volcano plots of the DEmRNAs between 3-years group and fresh group. **(D)** Heatmap showing the expression profile of DEmRNAs between the 3-years group and fresh group. **(E)** Statistically significant overlap between the upregulated mRNA lists from **(B,D)** determined by Fisher’s exact test (*p* < 2.2 × 10^−22^).

To compare with the fresh group, we identified 97 DEmRNAs in the 8-years group, and 87 DEmRNAs in the 3-years group (Supplementary Tables S2–S4). Among these DEmRNAs, 78 genes were upregulated and 19 were downregulated in the 8-years group, and in the 3-years group, 69 genes were upregulated and 18 were downregulated (Figures 2A,B). The expression patterns of these DEmRNAs were visualized by heatmap clustering analysis (Figures 2C,D). Interestingly, a significant overlap between the upregulated mRNAs sets of the 8-years group and the 3-years group (Figure 2E, *p* < 2.2 × 10^−22^) was detected, indicating that the two sets of upregulated mRNAs might be affected by the vitrification-warming procedure.

**FIGURE 3:**
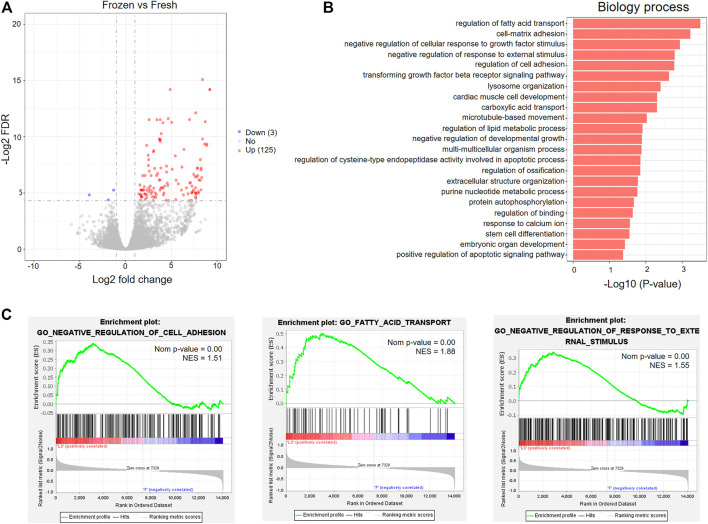
GO analysis of differentially expressed mRNAs between fresh embryos and vitrified-warmed embryos. **(A)** Volcano plots of the DEmRNAs between the frozen group and fresh group. **(B)** Significantly enriched GO terms in the biological process (BP) category associated with upregulated mRNAs in frozen embryos. **(C)** GSEA of upregulated mRNAs in frozen embryos.

To further investigate the effects of vitrification-warming on mRNA expression in human embryos, we directly compared the mRNA expression profiles of 8 vitrified-warmed embryos with those of fresh embryos. The distributions of the total expression of mRNAs in all embryos were nearly the same, as illustrated by the boxplot analysis (Supplementary Figure S1A). The correlation coefficient between the two embryos is about 0.965, which confirmed that the RNA-seq method is accurate and reproducible (Supplementary Figure S1B). We identified a total of 128 DEmRNAs between frozen embryos and fresh embryos (Supplementary Table S5), and found that 97.66% (125) of genes were upregulated in frozen embryos (Figure 3A and Supplementary Figure S1C). In order to investigate the functions of these DEmRNAs, Gene Ontology (GO) enrichment analysis was performed. Among the upregulated genes, a total of 23 significantly enriched biological process (BP) terms were identified, which were mainly associated with stress-related responses, metabolic processes, cell adhesion and apoptosis (Figure 3B and Supplementary Table S6). The GO analysis results were further confirmed and supplemented by GSEA through the evaluation of RNA-Seq data at the level of whole-genome-scale gene sets. GSEA using BP GO annotations showed that cell adhesion, fatty acid transport, response to external stimulus, fatty acid oxidation, hormone biosynthetic process, and small-molecule metabolic process were upregulated in the frozen group (Figure 3C and Supplementary Figure S2).

### Dynamic Expression of LncRNA

**FIGURE 4:**
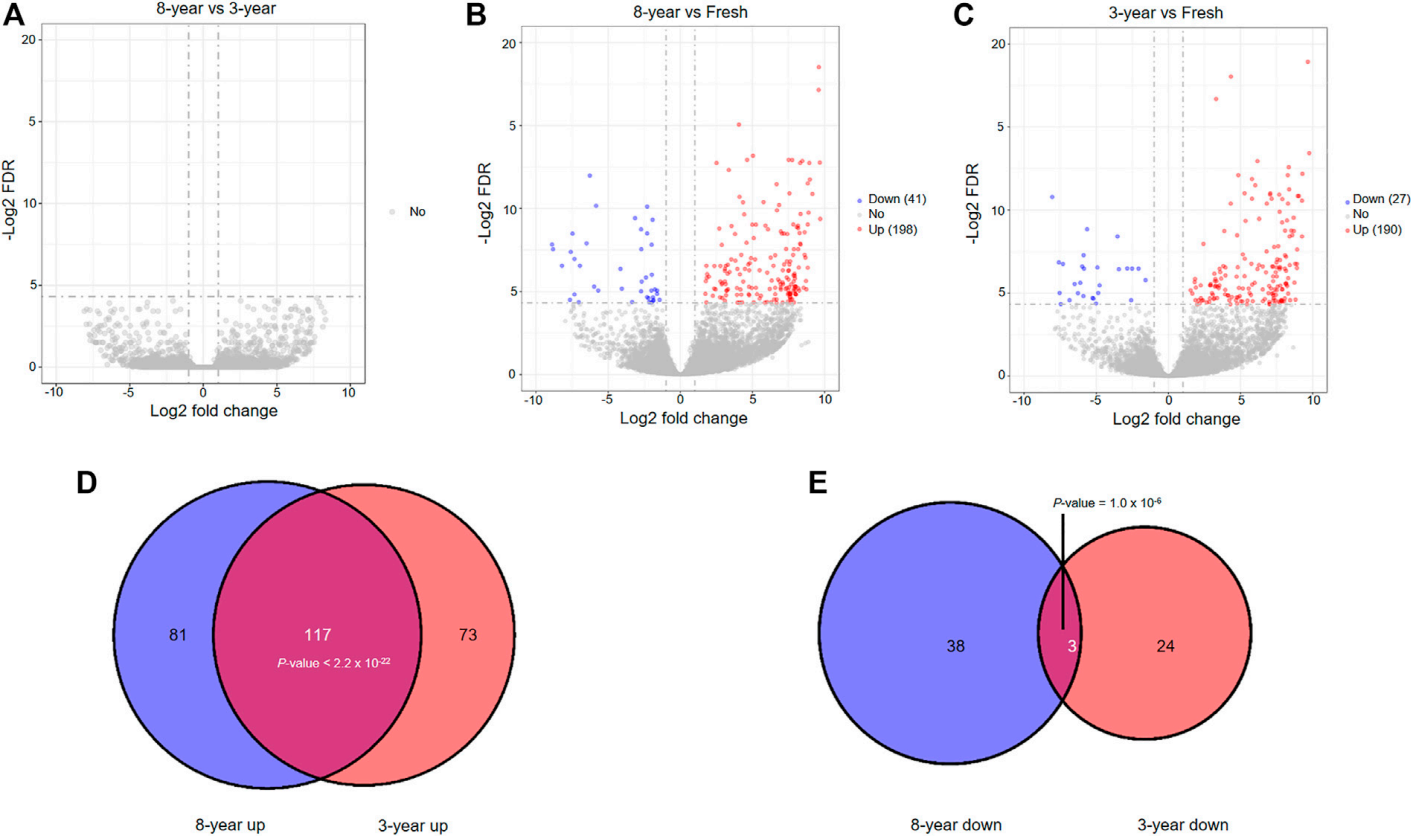
The analysis of differentially expressed lncRNAs among the three groups through pairwise comparisons. **(A)** Volcano plots of the DElncRNAs between the 8-years group and 3-years group. **(B)** Volcano plots of the DElncRNAs between the 8-years group and fresh group. **(C)** Volcano plots of the DElncRNAs between the 3-years group and fresh group. **(D)** Statistically significant overlap between the upregulated lncRNA lists from **(B,C)** determined by Fisher’s exact test (*p* < 2.2 × 10^−22^). **(E)** Statistically significant overlap between the downregulated lncRNA lists from **(B,C)** determined by Fisher’s exact test (*p* = 1.0 × 10^−6^).

We investigated whether the expression profiles of lncRNAs were affected by storage time after vitrification. The boxplot demonstrated that the distributions of the total expression of lncRNAs were nearly the same in all groups of embryos (Supplementary Figure S3A). Similar to the previously mentioned mRNAs, no significantly differentially expressed lncRNAs (DElncRNAs) were identified between the 8- and 3-years groups (Figure 4A). In addition, a total of 239 and 217 DElncRNAs were respectively identified in the 8-years (198 upregulated and 41 downregulated), and 3-years groups (190 upregulated and 27 downregulated), compared with the fresh group (Figures 4B,C, Supplementary Tables S7–S9). Interestingly, a significant overlap between the two upregulated lncRNAs sets in the two frozen groups was detected, and similar observation in the two downregulated lncRNAs sets (Figures 4D,E). In particular, 43.17% (117) of the total upregulated lncRNAs were shared between the two upregulated mRNAs sets, with a high statistical significance (*p* < 2.2 × 10^−22^), indicating that the upregulation of lncRNAs is mainly due to vitrification-warming procedures.

**FIGURE 5:**
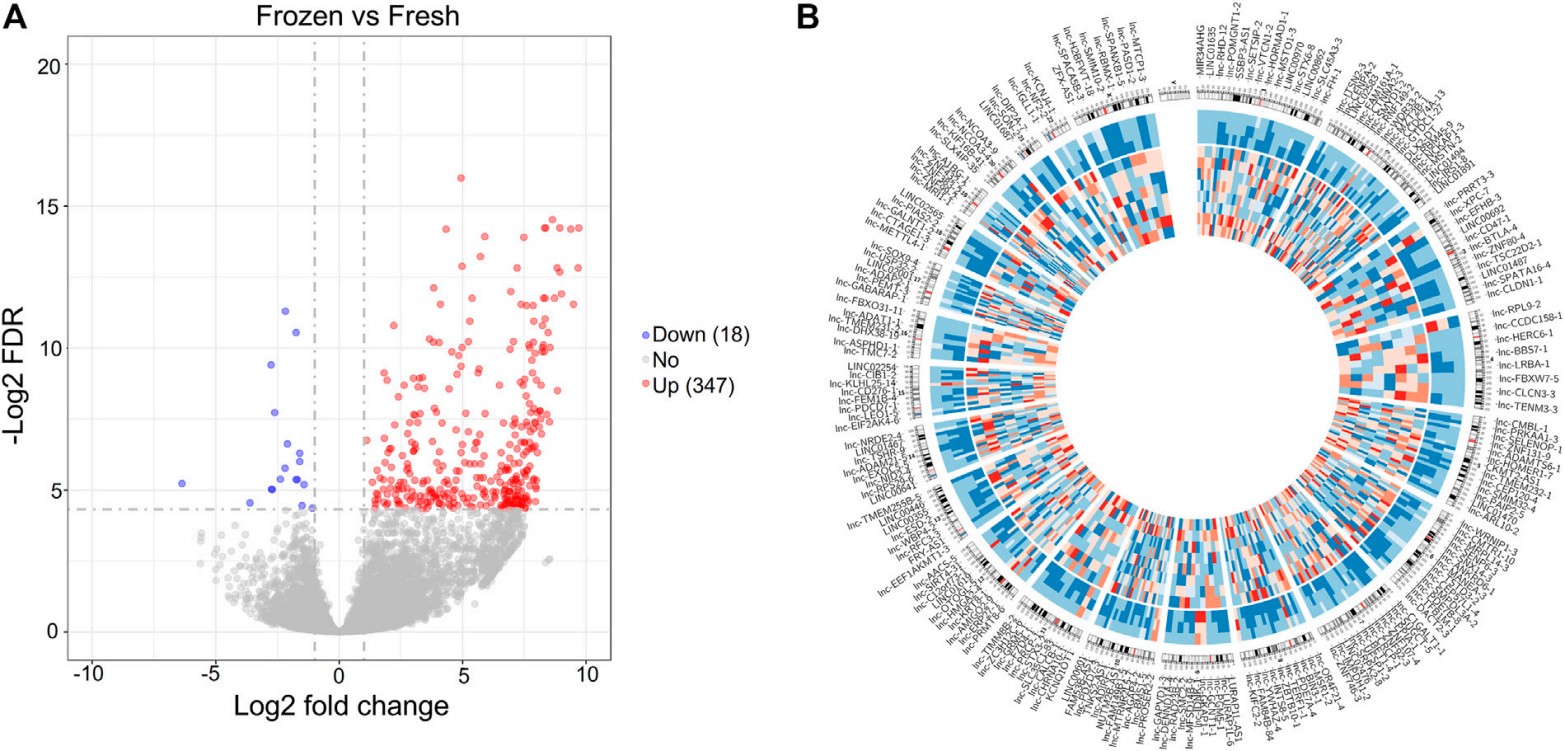
Differentially expressed lncRNAs between fresh embryos and vitrified-warmed embryos. **(A)** Volcano plots of the DElncRNAs between the frozen group and fresh group. **(B)** Circos diagram depicting the chromosomal distribution of DElncRNAs. The outside ring represents the chromosome map of the human genome, and the inside ring represents the expression heatmap of all the DElncRNAs.

Therefore, we compared the lncRNA expression profiles of the 8 frozen embryos with those of fresh embryos, and identified 365 DElncRNAs (Figure 5A and Supplementary Table S10). Among the DElncRNAs, 95.07% (347) of lncRNAs were upregulated in frozen group (Supplementary Figure S3C). The significantly altered lncRNAs were widely distributed on most chromosomes except for the Y chromosome, as shown in the Circos plot (Figure 5B).

### The *Cis* Regulation of DElncRNAs on Neighboring mRNAs

**FIGURE 6:**
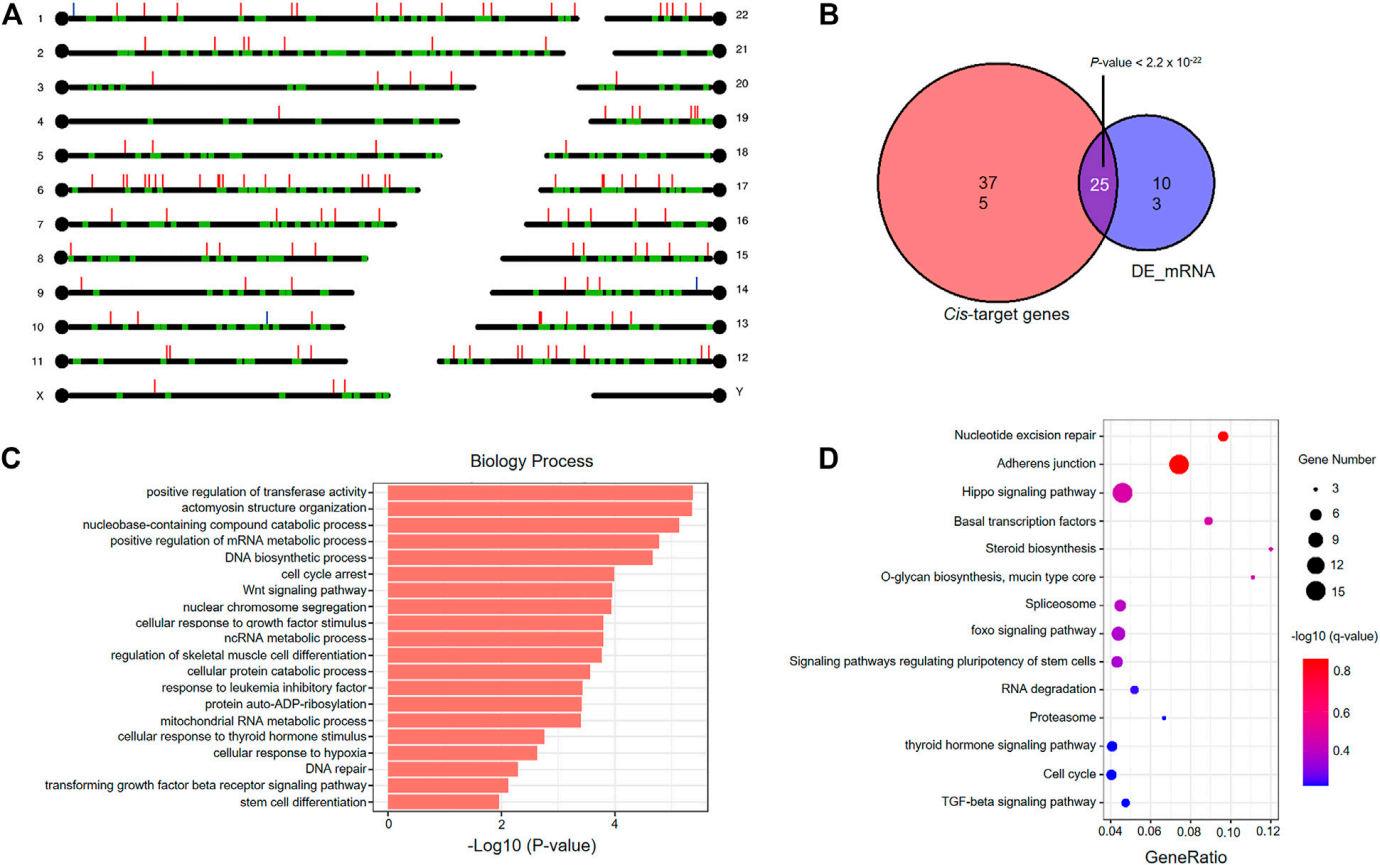
Functional analysis of *cis*-target genes of differentially expressed lncRNAs in vitrified-warmed embryos. **(A)** Loci of DEmRNAs and *cis*-target genes. Red lines represent upregulated lncRNAs; blue lines represent downregulated lncRNAs; green blots represent *cis*-target genes. **(B)** Statistically significant overlap between the *cis*-target mRNAs and DEmRNAs determined by Fisher’s exact test (*p* < 2.2 × 10^−22^). **(C)** Top 20 significantly enriched GO terms in the biological process (BP) category associated with *cis*-target mRNAs. **(D)** KEGG pathway analysis of *cis*-target mRNAs. The colors indicate significance, the size represents the number of genes enriching the corresponding annotation, and the GeneRatio is shown on the horizontal axis.

We searched the mRNAs nearest 10 kb cutoff for the 365 DElncRNAs derived from comparing fresh and frozen embryos, and identified 400 *cis*-target genes with 415 lncRNA-mRNA gene pairs (Figure 6A and Supplementary Table S11). Interestingly, we found a significant overlap between the *cis*-target genes and DEmRNAs derived from the comparison between vitrified-warmed embryos and fresh embryos, which means that DElncRNAs seems to be located close to DEmRNAs (Figure 6B). Enrichment analysis of GO terms and KEGG pathways were performed to identify *cis*-target genes to explore the biological function of DElncRNAs in vitrified-warmed embryos. The top 20 enriched BP GO terms included positive regulation of transferase activity, cell cycle, stress-related responses, DNA repair and metabolic process (Figure 6C and Supplementary Table S12). A total of 14 KEGG pathways were identified through enrichment analysis, including cell cycle, adherens junction, DNA repair, TFG-β and Hippo signaling pathways (Figure 6D and Supplementary Table S13). These enrichment results were consistent with the GO terms and GSEA gene sets associated with the upregulated mRNAs in the frozen embryos.

### Co-Expression Network Analysis of DElncRNAs and mRNAs

**FIGURE 7:**
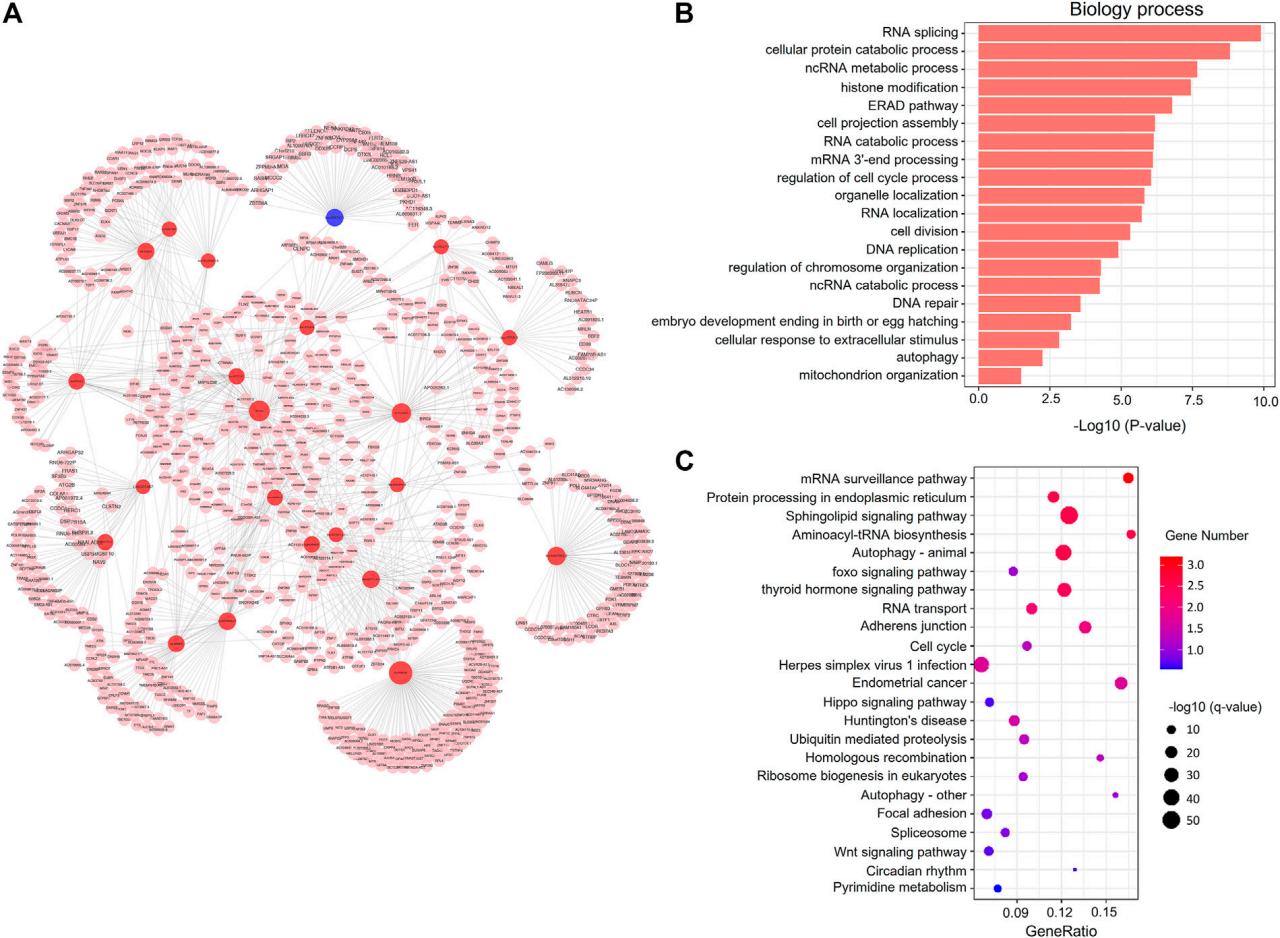
Co-expression network analysis of DElncRNAs and mRNAs. **(A)** The co-expression network. The red and blue circle nodes represent upregulated and downregulated lncRNAs in the frozen group, respectively. The pink circle nodes represent the *trans*-target genes. **(B)** Top 20 significantly enriched GO terms in the biological process (BP) category associated with *trans*-target mRNAs. **(C)** KEGG pathway analysis of *cis*-target mRNAs. The colors indicate significance, the size represents the number of genes enriching the corresponding annotation, and the GeneRatio is shown on the horizontal axis.

The results indicated that the whole network contained 1753 nodes and 2943 edges among 321 DElncRNAs and 1432 mRNAs (Supplementary Table S14). Based on the network topology measures of degree of centrality (DC), the most highly ranked hub lncRNAs were lnc-PAIP2-6 (DC = 110) and LINC02208 (DC = 85), followed by lnc-RAD23B-2 (DC = 71 and Supplementary Table S15). The network of lncRNAs (DC ≥ 25) combined with the target mRNAs is shown in Figure 7A.

We performed GO and KEGG enrichment analysis of the co-expressed genes of DElncRNAs, and found that significant BP GO terms were related to cell cycle, DNA repair, stress-related responses, and metabolic process (Figure 7B and Supplementary Table S16), which was similar to the GO results of the *cis*-target genes of DElncRNAs. Moreover, KEGG pathway analysis showed that several pathways were related to embryonic development, including cell adhesion, cell cycle, and the wnt and hippo signaling pathways (Figure 7C and Supplementary Table S17).

## Discussion

With the wider application of cryopreservation technology in ART, the number and time of cryopreserved embryos with vitrification have increased rapidly. Therefore, it is important to evaluate the safety of long-term cryopreservation on vitrified-warmed human embryos. This study demonstrated that storage time does not affect the mRNA and lncRNA expression profiles in vitrified-warmed human embryos.

It has been a longstanding debate regarding the influence of storage time on embryonic survival, and clinical outcomes. Although the successful birth of healthy babies from embryos after 20 years of storage has been reported (Kuwayama et al., 2005), the safety of long-term cryopreservation cannot be verified by a single case report. Previous report demonstrated that the survival rate of human embryos and the pregnancy rate decreased after several months of storage (Hamazaki et al., 2020). In addition, a recent study investigated the effect of extended storage time of vitrified cleavage-stage embryos on survival rate and clinical outcomes from 24,698 frozen transfer cycles, found that rates of implantation, clinical pregnancy, multiple pregnancy, and live births decreased with prolonged storage time (Li et al., 2020b). In contrast, many studies have shown that the length of cryopreservation time has no significant effects on the embryo survival rate or pregnancy outcomes of human embryos (Riggs et al., 2010; Liu et al., 2014; Li et al., 2017; Ueno et al., 2018).

During long-term cryopreservation, the quality of embryos may be affected mainly due to changes in temperature or pressure and radiation. In practical applications, repeated tank access to retrieve embryos and appropriate maintenance of storage tanks with liquid nitrogen influence storage conditions, which might have an impact on the developmental potential of embryos. Generally, the background radiation is 0.1 cGy/year. Frozen mouse embryos exposed to 200 cGy, which is equivalent approximately 2000 years of background radiation, showed that long-term storage does not interfere with the viability of cryopreserved embryos (Glenister and Lyon, 1986). However, the research on zebrafish embryos demonstrated that 0.37 cGy of radiation can induce the delay of hatching onset and that the expression of genes associated with apoptosis significantly changes at a total dose as low as 0.16 cGy of radiation (Hurem et al., 2017). Given the significant differences in physiology and anatomy between animals and humans, the results obtained from animal experiments could not be accurately applied to humans. To date, all clinical studies exploring the influence of long-term storage have focused on the survival rate and clinical outcomes. However, whether long-term cryopreservation results in transcriptomic alteration in human embryos remains unknown. In the present study, we found that different storage times did not alter either the mRNA or lncRNA expression profiles of the surviving vitrified-warmed human embryos. These results suggested the efficacy and safety of long-term storage of frozen embryos.

In addition to the finding of a stable transcriptome after long-term cryopreservation, our study also demonstrated that vitrification-warming procedures alter the expression of several genes. Significant overlaps between the gene lists of DEmRNAs or DElncRNAs were detected by comparing the 8-years group with the fresh group and the 3-years and fresh group, which also supported our hypothesis that the potential damage produced to vitrified-warmed embryos is only due to the vitrification-warming procedure. In this study, we only identified a total of 128 DEmRNAs between frozen embryos and fresh embryos, suggesting that the impact of vitrification-warming was minor. Interestingly, we found that 97.66% of DEmRNAs were upregulated genes in frozen embryos, which is consistent with the results obtained by Gutierrez-Castillo et al., who identified that 93.60% of the total DEmRNAs were upregulated genes in vitrified embryo (Gutierrez-Castillo et al., 2021). We speculated that because of the short duration of the vitrification procedure and recovery time after warming, there was no enough time for the degradation of RNAs or initiation of the mechanism of downregulation.

Many studies from mice, pigs, bovines, rabbits and humans have revealed that vitrification-warming can trigger cell stress-related responses (Lavara et al., 2011; Shaw et al., 2012; Kopeika et al., 2015; Gupta et al., 2017; Cuello et al., 2021; Gutierrez-Castillo et al., 2021). Based on the GO enrichment analysis and GSEA of upregulated mRNAs in viable vitrified-warmed embryos as compared to the fresh group, we also observed the enrichment of several stress-related responses, including negative regulation of cellular response to growth factor stimulus and response to external stimulus. Among genes involved in stress-related responses, Hic-5 is of special interest because of its role in TGF-β signaling. The TGF-β signaling pathway plays key roles in embryonic development, and regulates the transcription of genes that control cell proliferation, differentiation, and death (Wu and Hill, 2009). The upregulation of Hic-5 was found to suppress cell proliferation, and induce the cellular senescence and apoptosis by inhibiting the TGF-β pathway (Inui et al., 2012; Ruan et al., 2020). In addition, several metabolic pathways were activated after the vitrification-warming process, including the pathways of fatty acid transport, lipid and purine metabolism. The results are consistent with previous studies, which indicated that the upregulated genes were enriched in metabolic pathways in vitrified-warmed embryos (Aksu et al., 2012; Gupta et al., 2017). This finding supports the “quiet embryo” hypothesis, which proposes that the better viable embryos exhibit relatively lower metabolic activity (Leese, 2002). Supplementation with phenazine ethosulfate, a metabolic inhibitor of fatty acid synthesis, increased the developmental potential after vitrification (Sudano et al., 2011). Previous studies have demonstrated that vitrification can activate the apoptotic program (Shaw et al., 2012; Zhou et al., 2016). Here, we also identified the enrichment of positive regulation of the apoptotic signaling pathway. The Fis1 gene, encodes mitochondrial fission 1 protein, which was upregulated in vitrified-warmed embryos and is also involved in mitochondrial function. Mitochondria have important roles in fertilization and early embryonic development. A sufficient number of mitochondria are necessary to support the consumption of adenosine triphosphate (ATP), which occurs during the processes of early embryonic development (Van Blerkom et al., 1995; Ge et al., 2012; Murakoshi et al., 2013). The altered expression of Fis1 can trigger caspase-dependent cell death, by causing the release of cytochrome c from mitochondria (Alirol et al., 2006). In accordance with these results, we postulated that the vitrification-warming procedure might abnormally induce several stress-related pathways.

LncRNAs are involved in a variety of cellular processes, and certain lncRNAs have been identified to play important roles in early embryonic development (Durruthy-Durruthy et al., 2015; Hamazaki et al., 2015; Wang et al., 2016; Wang et al., 2018). In the present study, a total of 365 DElncRNAs were identified in vitrified-warmed embryos. Numerous studies have shown that lncRNAs achieve their function through regulation of target mRNAs, including *cis*- and *trans*-targets (Wilusz et al., 2009). Here, a total of 400 *cis*-target mRNAs within 10 kb upstream and downstream of the DElncRNAs were identified. Interestingly, we detected a significant overlap between the *cis*-target mRNAs and DEmRNAs derived from the comparison between the frozen group and fresh group, and found that 19.53% of DEmRNAs were *cis*-target mRNAs of DElncRNAs. Given that lncRNAs are more variable than mRNAs, especially under stress conditions, we postulated that vitrification-warming procedures might alter the expression of lncRNAs, and subsequently affect the transcription of a portion of mRNAs via the lncRNA-mediated regulation. In addition, a total of 1432 *trans*-target mRNAs were identified through co-expression network analysis. Consistent with the functional analysis of DEmRNAs in frozen embryos, the TGF-β signaling pathway, metabolic processes, stress-related responses, and cell adhesion is also identified, for the *cis*- and *trans*-target mRNAs. Moreover, other important biological processes (DNA repair and cell cycle arrest) and pathways (Hippo signaling pathway) were enriched for the target mRNAs. The activation of DNA repair and cell cycle arrest suggested that vitrification-warming triggered DNA damage and transient stress, and whether the molecular differences can be eliminated in the subsequent embryo culturing needs to be tested further. The Hippo signaling pathway has been demonstrated to play a central role in the specification of the first cell fates during early embryonic development, and abnormal activation might induce apoptosis and then produce cell death (Yu et al., 2015; Peng et al., 2019).

The limitations of this study are worth mentioning. Experimental validation in a larger-scale cohort of samples would be beneficial, although the stringent criteria for DEmRNA and DElncRNA identification (FDR <0.05, > 2-fold change) were used in the present study. Moreover, further studies are warranted to verify whether long-term cryopreservation has an impact on other molecular mechanisms, such as epigenetic modification.

## Conclusion

This is the first study exploring the effect of storage time on mRNA and lncRNA expression profiles in human embryos after vitrification. Our results indicated that long-term cryopreservation does not affect the transcriptomes of human embryos at the single cell level. Furthermore, the effect of vitrification-warming on human embryos is minor in terms of the small number of DEmRNAs and DElncRNAs. However, further research is needed to verify whether these changes in gene expression have an impact on embryos.

## Data Availability

The raw data for this study can be found in the NCBI public database at this URL link: https://trace.ncbi.nlm.nih.gov/Traces/sra/?study=SRP355713.
