# Supplementary figures and images for: Long-Term Storage Does Not Affect the Expression Profiles of mRNA and Long Non-Coding RNA in Vitrified-Warmed Human Embryos
Source: Front Genet. 2022 Feb 1;12:751467. doi: 10.3389/fgene.2021.751467 (PMC8844023; doi:10.3389/fgene.2021.751467)

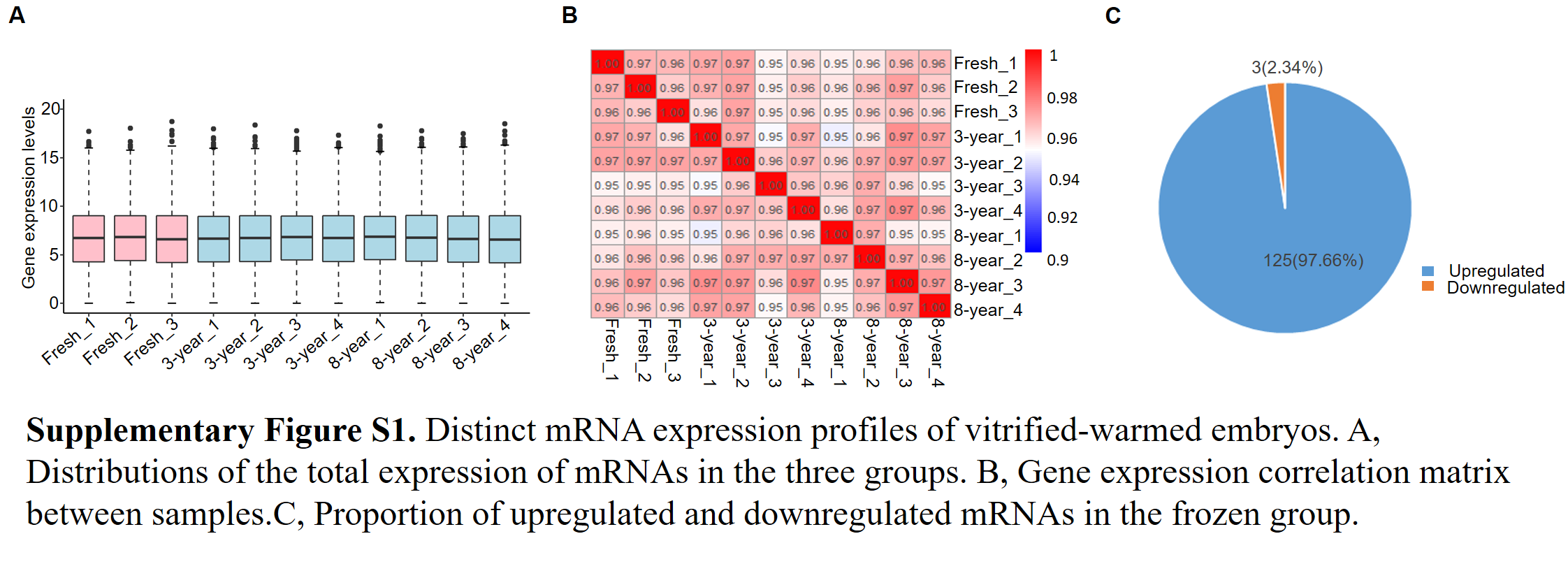

Supplement: Supplementary file 1 [file DataSheet1.zip › Supplemental Figures/Supplementary Figure S1.tif]

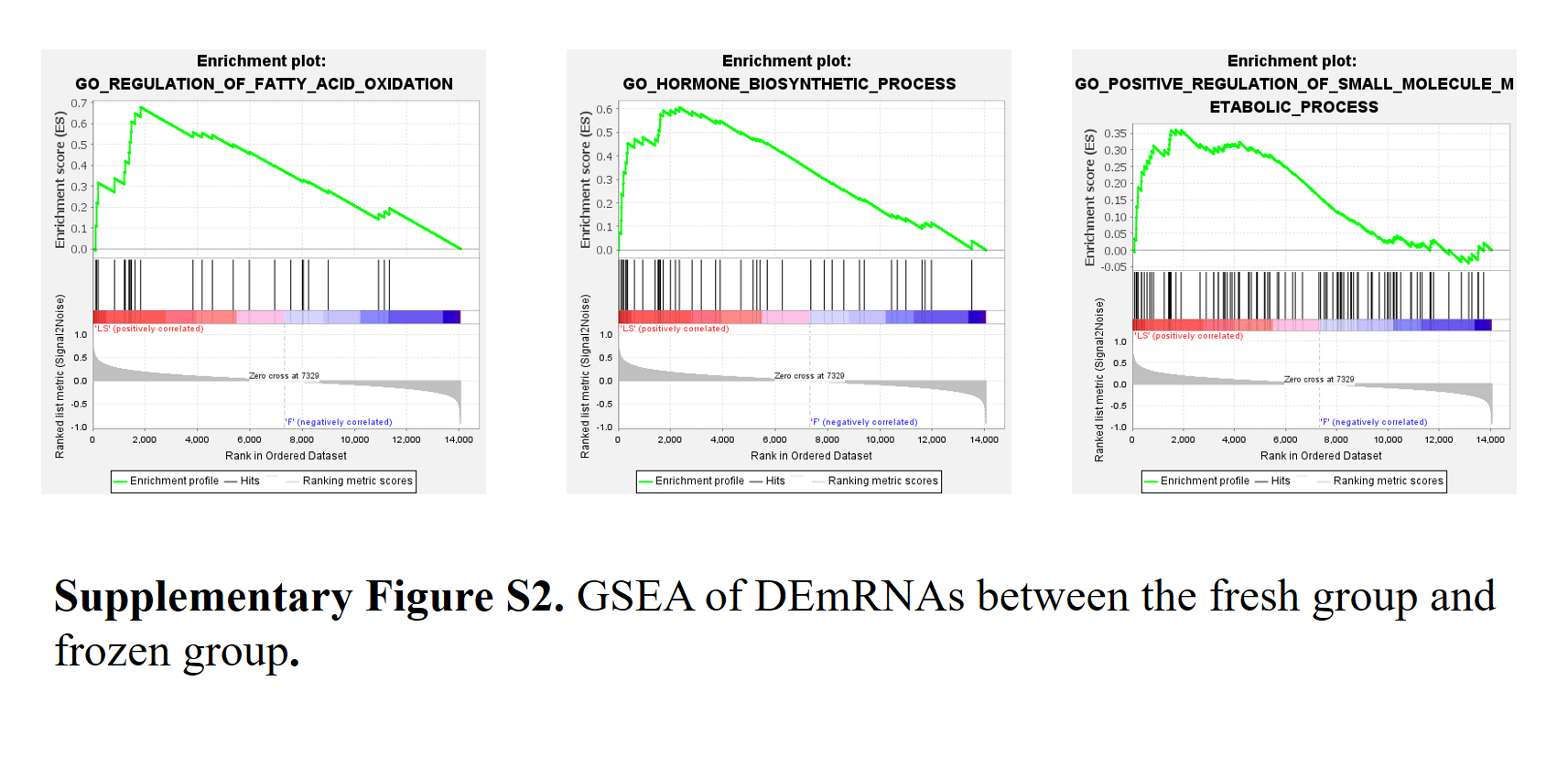

Supplement: Supplementary file 1 [file DataSheet1.zip › Supplemental Figures/Supplementary Figure S2.tif]

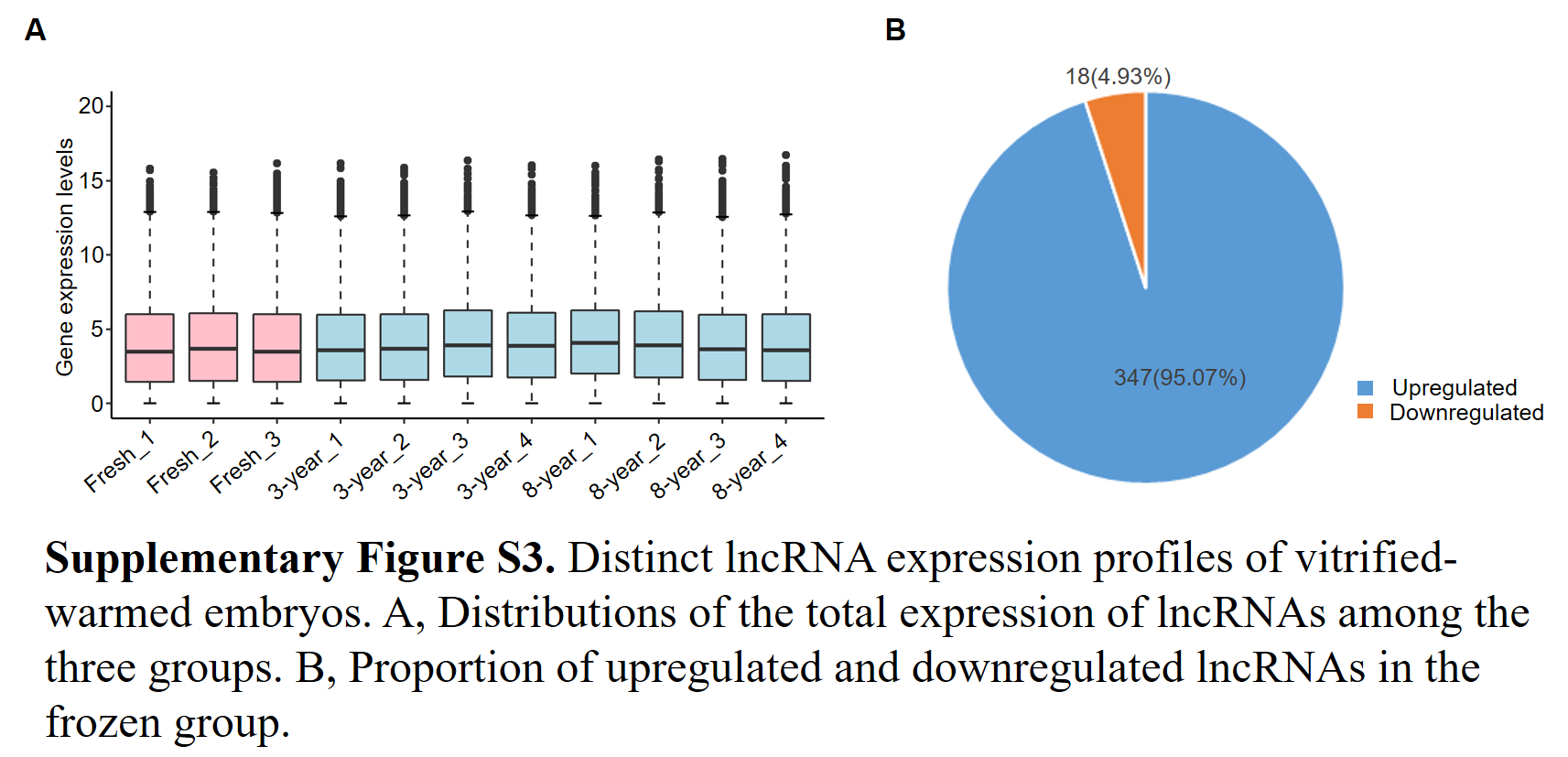

Supplement: Supplementary file 1 [file DataSheet1.zip › Supplemental Figures/Supplementary Figure S3.tif]
