# Supplementary material for: Long-Term Storage Does Not Affect the Expression Profiles of mRNA and Long Non-Coding RNA in Vitrified-Warmed Human Embryos
Source: Front Genet. 2022 Feb 1;12:751467. doi: 10.3389/fgene.2021.751467 (PMC8844023; doi:10.3389/fgene.2021.751467)
Supplement: Supplementary file 1 [file DataSheet1.zip › Supplementary Tables/Supplementary Table 1.doc]

**Table S1 Summary of the sequencing qualities and reads mapping.**

| **Sample_ID** | **Raw reads** | **Clean Reads** | **Q30 (%)** | **Mapped reads** | **Rate (%)** |
| --- | --- | --- | --- | --- | --- |
| Fresh_1 | 40,340,432 | 35,641,414 | 93.55 | 34,515,145 | 96.84 |
| Fresh_2 | 44,567,604 | 4,172,6514 | 93.97 | 40,666,661 | 97.46 |
| Fresh_3 | 41,939,132 | 39,374,022 | 93.90 | 38,456,607 | 97.67 |
| 3-year_1 | 42,762,414 | 34,997,364 | 93.29 | 33,796,954 | 96.57 |
| 3-year_2 | 49,993,680 | 48,968,860 | 95.54 | 41,662,706 | 85.08 |
| 3-year_3 | 43,661,640 | 41,926,464 | 95.35 | 39,494,729 | 94.20 |
| 3-year_4 | 43,014,780 | 41,821,028 | 95.7 | 39,696,520 | 94.92 |
| 8-year_1 | 44,540,308 | 43,418,984 | 95.23 | 41,434,736 | 95.43 |
| 8-year_2 | 46,569,600 | 36,193,998 | 92.49 | 34,684,708 | 95.83 |
| 8-year_3 | 38,297,160 | 36,897,222 | 93.93 | 35,022,843 | 94.92 |
| 8-year_4 | 51,531,134 | 50,330,418 | 95.63 | 47,874,294 | 95.12 |
